# Accumulation of mechanical forces in tumors is related to hyaluronan content and tissue stiffness

**DOI:** 10.1371/journal.pone.0193801

**Published:** 2018-03-21

**Authors:** Chrysovalantis Voutouri, Triantafyllos Stylianopoulos

**Affiliations:** Cancer Biophysics Laboratory, Department of Mechanical and Manufacturing Engineering, University of Cyprus, Nicosia, Cyprus; University of Michigan, UNITED STATES

## Abstract

Hyaluronan is abundant in the extracellular matrix of many desmoplastic tumors and determines in large part the tumor biochemical and mechanical microenvironment. Additionally, it has been identified as one of the major physiological barriers to the effective delivery of drugs to solid tumors and its targeting with the use of pharmaceutical agents has shown to decompress tumor blood vessels, and thus improve tumor perfusion and efficacy of cytotoxic drugs. In this study, we investigated the contribution of hyaluronan to the accumulation of mechanical forces in tumors. Using experimental data from two orthotopic breast tumor models and treating tumors with two clinically approved anti-fibrotic drugs (tranilast and pirfenidone), we found that accumulation of growth-induced, residual forces in tumors are associated with hyaluronan content. Furthermore, mechanical characterization of the tumors revealed a good correlation of the accumulated forces with the elastic modulus of the tissue. Our results provide important insights on the mechano-pathology of solid tumors and can be used for the design of therapeutic strategies that target hyaluronan.

## Introduction

Solid tumors often stiffen as they grow at the expense of the surrounding host tissue. Tissue stiffening is caused by an increase in the amount of cancer cells, stromal cells and the extracellular matrix constituents, mainly collagen and hyaluronan. As a tumor grows, cancer cells divide faster than normal cells, the tumor becomes stiffer and displaces the surrounding normal tissue, which allows tumor progression. Therefore, tumor growth is associated with the generation of mechanical stresses that affect tumor progression and response to treatment in several ways [1–5]. The three tissue-level types of solid stress (i.e., stress of the solid components) that are developed in tumors are: i) stress applied externally to the tumor by the host tissue, ii) growth-induced (or residual) stress, which is accumulated in the tumor and remains even if the tumor is excised, and iii) swelling stress owing to the swelling behavior of the hyaluronan [6].

**Fig 1:**
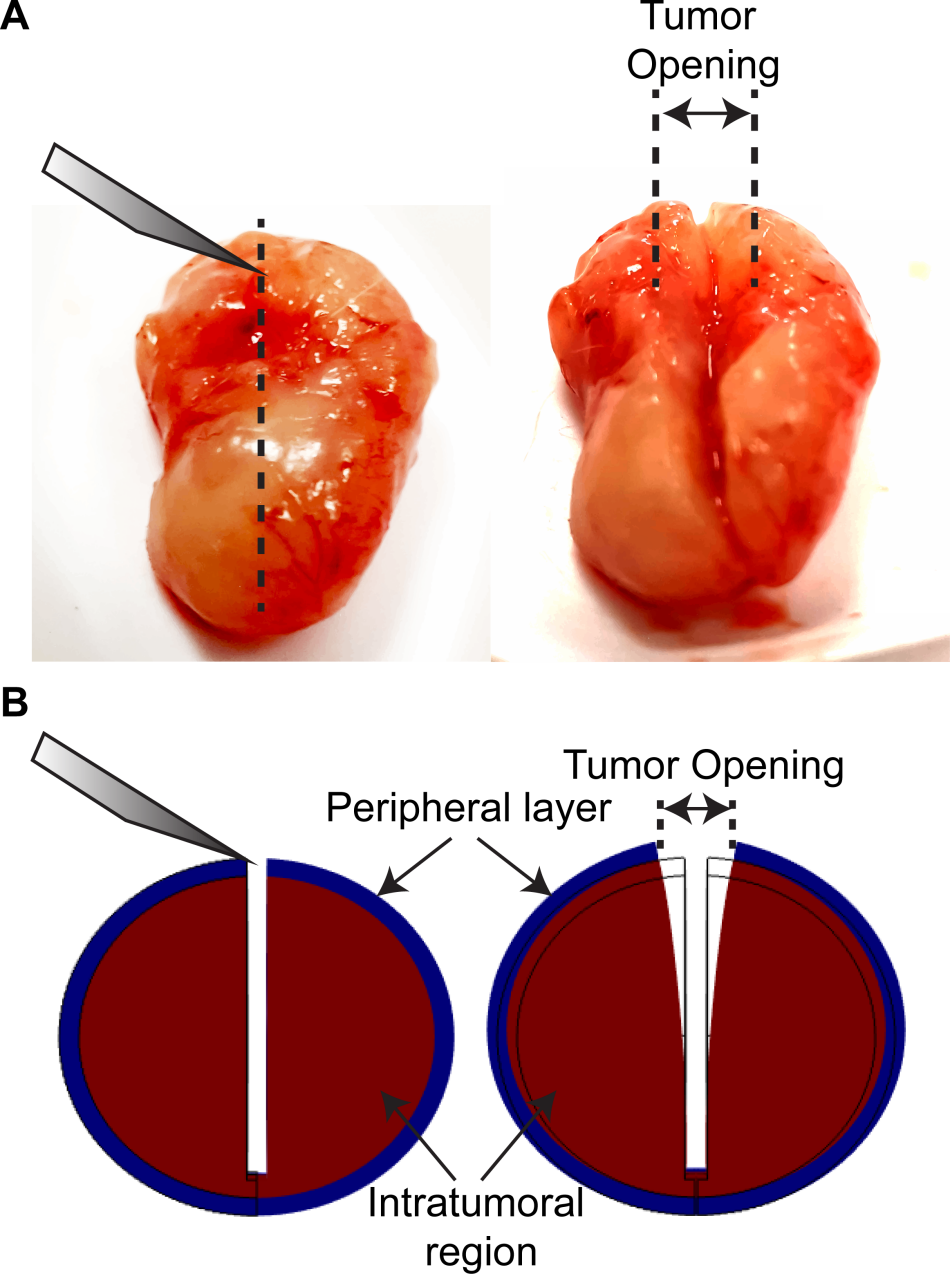
Schematic of tumor opening experiment and calculations. **A**: Typical experimental procedure showing the tumor before and after the cut has been made. The measured tumor opening appears in the figure. **B**: Representative computational results in the beginning and at the end of the simulation. In the model, the tumor consists of two domains, the tumor and a peripheral layer with thickness 5% of the tumor diameter. The simulations were used for the calculation of the growth-induced stress from the measured displacement/opening of the tumor.

**Fig 2:**
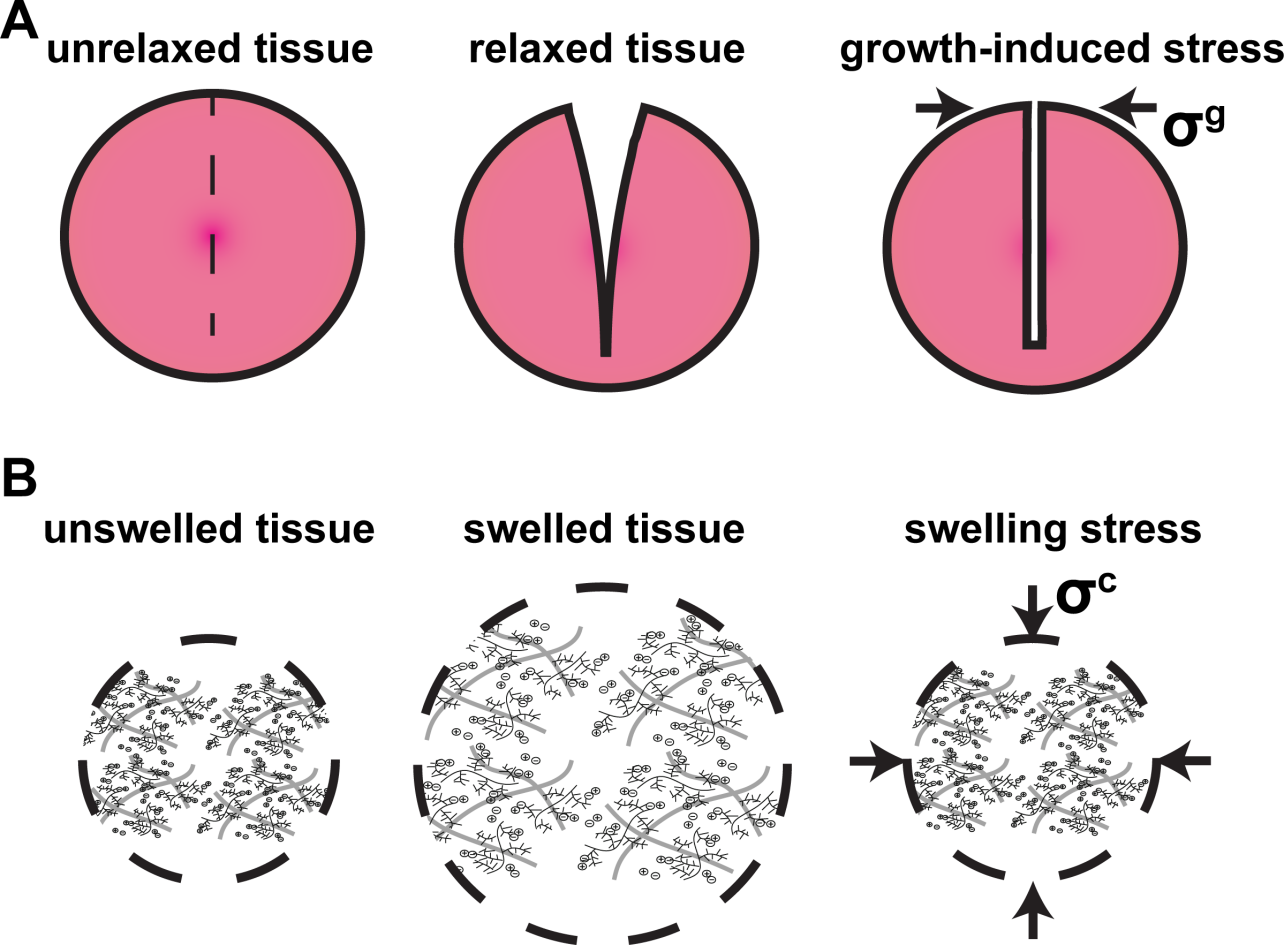
Schematic of growth-induced stress and swelling stress. **A:** Growth-induced stress, **σ**^g^, is equal to the stress required to close the tumor after the tumor relaxes and the stress is released. **B:** Swelling stress, **σ**^**c**^, is the stress required to compress tumor to initial radius from swelled tissue condition.

Growth-induced stress is believed to be generated owing to the fact that solid stresses in tumors are contained within viscoelastic structural components of the tissue, and thus, some stresses are maintained even if a tumor is excised. Mechanical interactions that could generate growth-induced stress could be the stretching and remodelling of extracellular fibers by cancer cells or activated cancer-associated fibroblasts (CAFs) and the hydration of hyaluronan that resists compressive forces within the tumor. Growth-induced stress in tumors has been quantified in previous research by excising tumors and making a cut along their long axis at about ~80% of their thickness [7]. The cut causes retraction of the surface and bulging of the interior of the tumor. These deformation modes are owing to the relaxation of the growth-induced stress and result in a measurable tumor opening (Figs 1 and 2A) [7]. The bulging of the tumor interior is an evidence of compressive growth-induced stress, while the retraction of the surface is an evidence of tensile growth-induced stress at the tumor periphery that balances the compression at the interior of the tumor [6]. We have shown that selective depletion of CAFs, collagen or hyaluronan can significantly decrease growth-induced stress levels [7]. We have also found that this stress is accumulated in the tissue during tumor growth [8].

Swelling stress is developed owing to the negatively charged hyaluronan chains that exert repulsive, “charge-to-charge”, electrostatic forces on each other as they are closely packed in the dense tumor extracellular matrix, causing swelling of the tissue (Fig 2B) [9–12]. Swelling stress can be measured by placing a tissue specimen in a confined chamber and measuring with a load cell the force that is developed when the electrolyte concentration of the chamber is changed to hypotonic, the tissue tries to swell but the chamber prevents its expansion [9,11]. Selective depletion of hyaluronan decreases tissue swelling, while depletion of collagen increases swelling suggesting that collagen fibers, which strongly support tensile loads, are stretched during tumor swelling and thus, resist further swelling of the tumor. Interestingly, using different types of breast and pancreatic cancers as well as soft tissue sarcomas, we found that swelling stress correlates extremely well with the ratio of the hyaluronan to the collagen content through a linear relation (Eq 4 in Materials and Methods) [11].

Even though both collagen and hyaluronan content contribute to the accumulation of growth-induced stresses in tumors, an analysis of which of the two components is more closely related to this stress type is lacking in the literature. Additionally, a comparison between growth-induced and swelling stress in the same tumors has not been performed thus far. Importantly, pertinent studies for engineered tissues and blood vessels show a correlation between growth-induced and swelling stress [13–18]. Furthermore, data from our group have showed that treatment with the anti-fibrotic drugs tranilast or pirfenidone reduced hyaluronan content and hence the growth-induced and swelling stresses in tumor-treated samples [11,17,18]. These data further suggest a potential correlation between the two stress types.

To this end, the objective of this study is the measurement of growth-induced stress in tumors and the investigation of its relation to collagen and hyaluronan content as well as its comparison to swelling stress. First, we experimentally measure the tumor opening and elastic modulus in mock-treated tumors and tumors treated with tranilast or pirfenidone. Combining these experimental measurements with mathematical modelling, we predict the growth-induced stress levels for each tumor following our previously developed methodology [7]. Immunofluorescence analysis of the compared groups is also employed to assess the reduction of collagen and hyaluronan components. This is confirmed by measuring the area fraction of collagen and hyaluronan. Swelling stress is predicted by the ratio of hyaluronan to collagen content [11]. Finally, the inter-dependence of tumor opening, growth-induced stress, swelling stress, tumor composition and elastic modulus is explored.

## Material and methods

### Cell culture

MCF10CA1a human breast cancer cells (Karmanos Cancer Institute, Detroit, MI, USA) and 4T1 mouse mammary carcinoma cells (ATCC) were employed.

### Drugs and reagents

Tranilast (Rizaben®, Kissei Pharmaceutical, Japan) was solubilized with 1% NaHCO_3_ followed by heating at 70^°^C for 1 h (33.3mg/ml) [17,19,20]. Pirfenidone (Esbriet®, Roche Pharmaceuticals, Switzerland) was solubilized with sterile water followed by warming at 60^°^C for 30min [18]. We have previously showed that these drugs can reduce levels of hyaluronan and collagen type I in murine breast tumor models [17,18].

### Animal tumor models and experimental protocols

Orthotopic breast tumor models were generated by implantation of 5×10^5^ MCF10CA1a cells into the mammary fat pad of 6-week old female CD1 nude immunodeficient mice. Orthotopic syngeneic models for murine mammary tumors were generated by implantation of 10^5^ 4T1 mouse mammary cancer cells into the mammary fat pad of 6-week old BALB/c female mice. Pirfenidone and trainlast were administered orally once a day starting from day 4 post-implantation and for a period of 21 days. To measure alterations in the tumor microenvironment, right before the end of the experiment, animals were anesthetized by i.p. injection of avertin (200mg/kg) and they were sacrificed via CO_2_ inhalation. Subsequently, tumors were excised for measurement of mechanical properties and/or histological analysis. All in vivo experiments were conducted in accordance with the animal welfare regulations and guidelines of the Republic of Cyprus and the European Union (European Directive 2010/63/EE and Cyprus Legislation for the protection and welfare of animals, Laws 1994–2013) under a license acquired and approved (No CY/EXP/PR.L1/2014) by the Cyprus Veterinary Services committee, the Cyprus national authority for monitoring animal research for all academic institutions. All surgery was performed under Tribromoethanol (avertin) anesthesia, and all efforts were made to minimize suffering.

### Fluorescent immunohistochemistry

Following the tumor opening experiment, part of the tumor was taken for histological analysis. Tumor specimens were fixed and embedded in optimal cutting temperature compound. Transverse 40μm-thick tumor sections were produced and immunostained with antibodies against collagen I (ab4710, Abcam), CD31 (MEC13.3, BD Biosciences) and hyaluronan (ab53842, Abcam), counterstained with 4',6-diamidino-2-phenylindole (DAPI, Vector Labs). Antigens were detected using appropriate secondary fluorescent antibodies. Images were analyzed based on the area fraction of positive staining. The area fraction analysis was performed automatically using a previously developed in-house code to avoid any bias [7]. Five different sections per tumor (from the interior and the periphery) at ×10 magnification were taken and analyzed keeping the analysis settings and thresholds the same for all tumors.

### Tumor opening measurements

When tumors reached a size of ~ 1cm in diameter, they were excised and their three dimensions were measured (width, height, thickness). For the tumor opening measurement, a cut was made along the tumor's longest axis (∼80% of its thickness). The tumor was then allowed to relax for 10min to allow for any transient, poroelastic response to vanish and the opening at the surface of the tumor was measured as shown in Fig 1A [7]. During the experimental procedure the tumors remained hydrated by addition of phosphate buffer saline (PBS).

### Mechanical testing measurements for calculation of elastic modulus

The unconfined compression experimental protocol was employed for the measurement of the elastic modulus. Following the tumor opening experiment a part of the tumor 3×3×2mm (length × width × thickness) was selected and tumor specimens were loaded on a high precision mechanical testing system (Instron, 5944, Norwood, MA, USA). Stress-strain experiment was performed to a final strain of 30% with a strain rate of 0.05mm/min, the lowest rate the system can apply to avoid any poroelastic effects. The elastic modulus was calculated from the slope of the stress-strain curve in the range of 25–30% strain. A representative stress-strain curve is shown in Figure A in S1 File.

### Mathematical model for calculation of growth-induced stress

To calculate the growth-induced stress from the tumor opening experiment a mathematical model was employed to simulate the opening experiment and convert the measured strain to stresses following our previous methodology (Fig 1B) [7]. Notice that this methodology was developed to quantify a residual stress that is stored during growth. Growth-induced stress can also be purely due to cells and can be measured with methodologies that employ multicellular spheroids growing in permeable elastic capsules [21]. Here we present a summary of our mathematical model.

The tumor mechanical properties were taken to be isotropic and governed by the compressible neo-Hookean constitutive equation with strain energy density function given by [7,22–25]

$$W=0.5\mu \left(-3+II_{1}\right)+0.5\kappa {\left(-1+J\right)}^{2}$$ (1)

where *μ* is the shear modulus, *κ* is the bulk modulus, *J* is the determinant of the deformation gradient tensor **F**, and *II*_1_ is the second invariant of the right Cauchy-Green deformation tensor, evaluated from **F**. The values of the shear and bulk modulus that was used in the simulations were calculated for each tumor specimen separately using the experimentally measured elastic modulus and assuming a Poisson's ratio of 0.4 [8,22].

#### The momentum balance equation

The total stress of the tissue was take to be the sum of solid (elastic and growth-induced) stress [8,11,22,23,26]. The quasi-static linear momentum balance equation suggests that the divergence of the total stress tensor should equal zero:

$$\nabla ∙(\sigma^{total})=\nabla ∙(\sigma^{s}-\sigma^{g})=0$$ (2)

where **σ**^*s*^ is the elastic solid stress tensor and **σ**^*g*^ is the growth-induced stress tensor.

The elastic Cauchy stress tensor, **σ**^*s*^, was calculated by the strain energy density function as:

$$\sigma^{s}=J^{-1}F\frac{\partial W}{\partial F^{T}}$$ (3)

The growth-induced stress tensor **σ**^*g*^ represents the stress that was released in the tumor during the opening experiment. Following our assumption for isotropic growth, **σ**^*g*^ was assumed to be an isotropic tensor.

### Model parameters and formulation

For each tumor specimen and based on its three dimensions and elastic modulus, we developed a 3D Finite Elements model using the Solid Mechanics Physics in COMSOL (COMSOL, Inc., Burlington, MA, USA) (Figure B in S1 File) to solve the model equations. The number of finite elements comprising the model was 526,467. A mesh-independence analysis of the solution of the model is presented in Figure D in S1 File. The model has two different regions, the first is the intratumoral region, where the compressive stress at equilibrium is equal to growth-induced stress. The second region is the collagenous peripheral layer that forms a capsule surrounding the tumor. The capsule was assumed to be free from any growth-induced stress component and to be stiffer than the inner region of the tumor (i.e., higher shear modulus) [7]. We measured the thickness of the peripheral layer from histological analysis of the tumors and found it to range from 1.5% to 15% relative to the thickness of the tumor (Figure E in S1 File). For the baseline calculations we took the modulus of the peripheral layer to be 10 times stiffer than the tumor and the thickness of the layer was taken to be 10% of the thickness of the tumor [7]. To calculate the range of the compressive growth-induced stress on each tumor sample, we assumed for the modulus and the thickness of the peripheral layer to vary from 1.5 to 20 and from 1.5% to 15% relative to the modulus and thickness of the tumor, respectively. This range is shown in the figures using bars.

The simulation took into account the state of the tumor right after the cut has been made, modeling the bulging of the intatrumoral region and the opening of the tumor. We run simulations for each tumor specimen considering its specific dimensions and mechanical properties and our goal was for the model to match the experimentally measured tumor opening. To achieve this we varied the value of the isotropic growth-induced stress, **σ**^*g*^ (Eq 2) so that the difference between the experimentally measured and computed tumor opening to be less than 0.01mm.

Because of symmetry we performed our analysis in one-quarter of the domain. The boundary conditions employed are illustrated in Figure B in S1 File.

### Calculation of swelling stress

A detailed description of the calculation of swelling stress can be found in [11]. Briefly, a tumor specimen was placed in a confined chamber and compressed with a piston to 10% strain. Subsequently, it was allowed to relax completely so that any poroelastic response would vanish. Then, NaCl solution of specified concentration was added to the chamber to change the tonicity of the tissue, keeping the position of the piston fixed. Hypotonic solutions caused the swelling of the tissue and the force developed on the fixed piston was measured with a load cell. In previous research, we found a linear relationship to correlate extremely well the swelling stress to the ratio of hyaluronan to collagen area fraction [11]. This relationship is

[swellindstress]=4.09[HA/collagen]−2.057, (4)

where [HA/collagen] is the ratio of the area fraction of hyaluronan to collagen. Notice that this equation applies to the most hypotonic solution (0.001M NaCl) employed in [11] and it was found with the use of mathematical modeling to be the same to the *in vivo* situation.

### Statistical analysis

The experimental data are presented as means with standard errors. We performed an analysis of variance, or ANOVA/Tukey-Kramer paired analysis using the software program GraphPadPrism (6.0 for Windows; GraphPad Prism Software Inc., San Diego, CA) at the 95 percent confidence interval (CI).

## Results

### Experimental measurements for control and treated breast tumors

**Fig 3:**
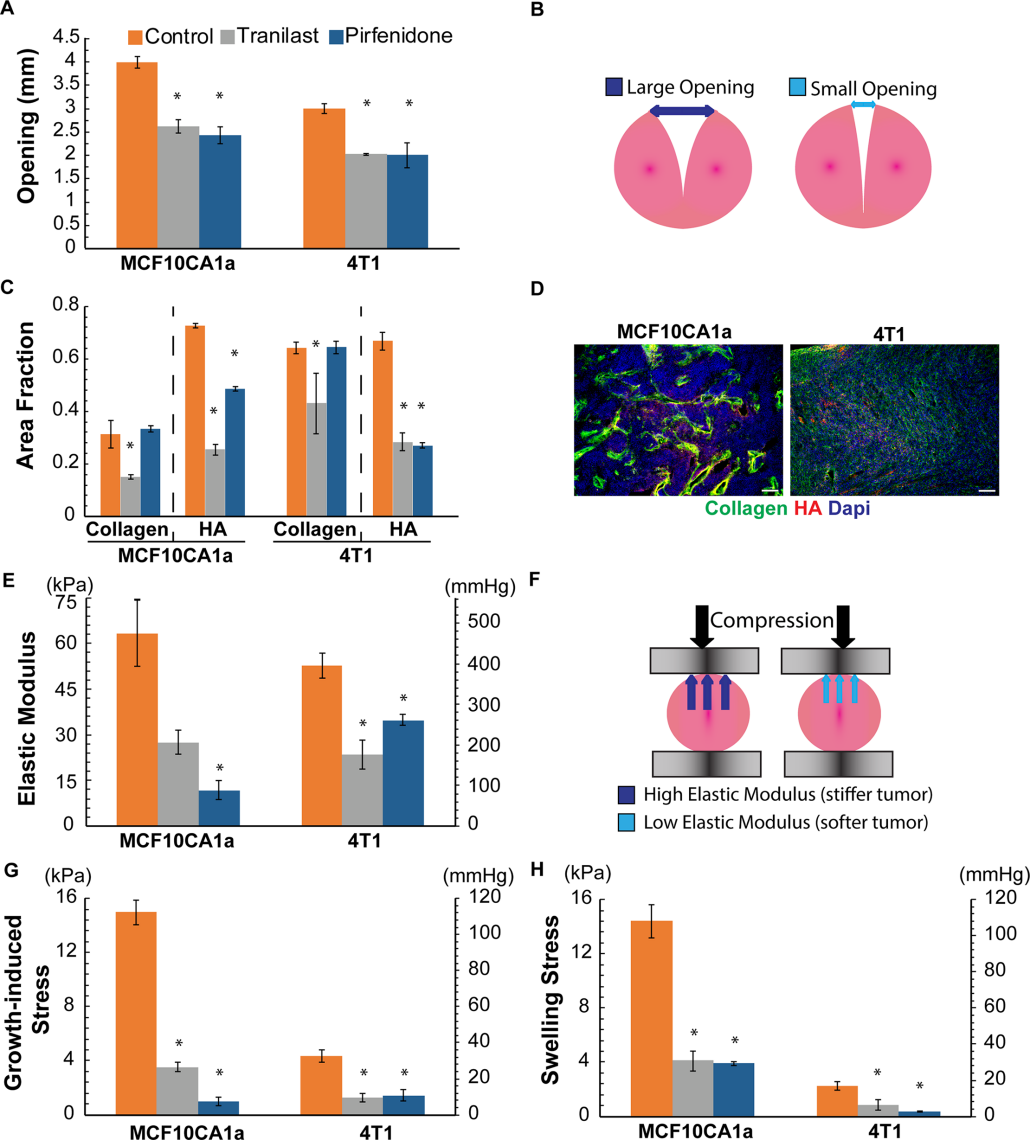
Experimental measurements and calculations for MCF10CA1a and 4T1 tumors. **A**: Tumor opening experimental data for the control and the two treated groups. **B**: Schematic of the tumor opening measurement, **C**: Area fraction quantification of collagen and hyaluronan (HA), **D**: Representative immunofluorescent images for collagen, HA and DAPI (scale bar:100μm). **E**: Elastic Modulus experimental data for the two tumors and the different groups employed. **F**: Schematic of mechanical measurements, high and low modulus is associated with stiffer and softer tumors, respectively, **G**: Growth-induced stress calculated using mathematical modeling, **H**: Swelling stress calculated from the ratio of hyaluronan to collagen content using Eq 4. Error bars represent the standard error.

Orthotopic MCF10CA1a and syngeneic 4T1 tumors were treated with tranilast (200mg/kg) or pirfenidone (500mg/kg) orally on a daily basis, while there was also mock-treated groups as control. When tumors were excised, they were cut along their longest axis and allowed to relax before measuring the tumor opening (Figs 1, 3A and 3B). In agreement with previous studies [7], tumor opening was higher in the control tumors compared to the treated groups, exhibiting higher levels of growth-induced stress. The results of the immunofluorescence staining and mechanical testing analyses are presented in Fig 3C–3F. More specifically, tranilast treatment reduced collagen and hyaluronan content in both tumor types. Pirfenidone even though it has been shown to affect both ECM components [11,18], in this study it reduced only hyaluronan levels without affecting collagen (Fig 3C and 3D and Figure E in S1 File). Modulation of the tumor extracellular matrix resulted in softer tumors of lower elastic modulus compared to the control groups with the exception of tranilast in MCF10CA1a tumors (Fig 3E and 3F). Subsequently, mathematical analysis of the experimental measurements was employed for the assessment of the growth-induced and swelling stress (Fig 3G and 3H). Both stress types were reduced for the treated groups compared to the control. Interestingly, the levels of growth-induced and swelling stress in the corresponding groups were similar.

### Tumor opening increases with hyaluronan area fraction

**Fig 4:**
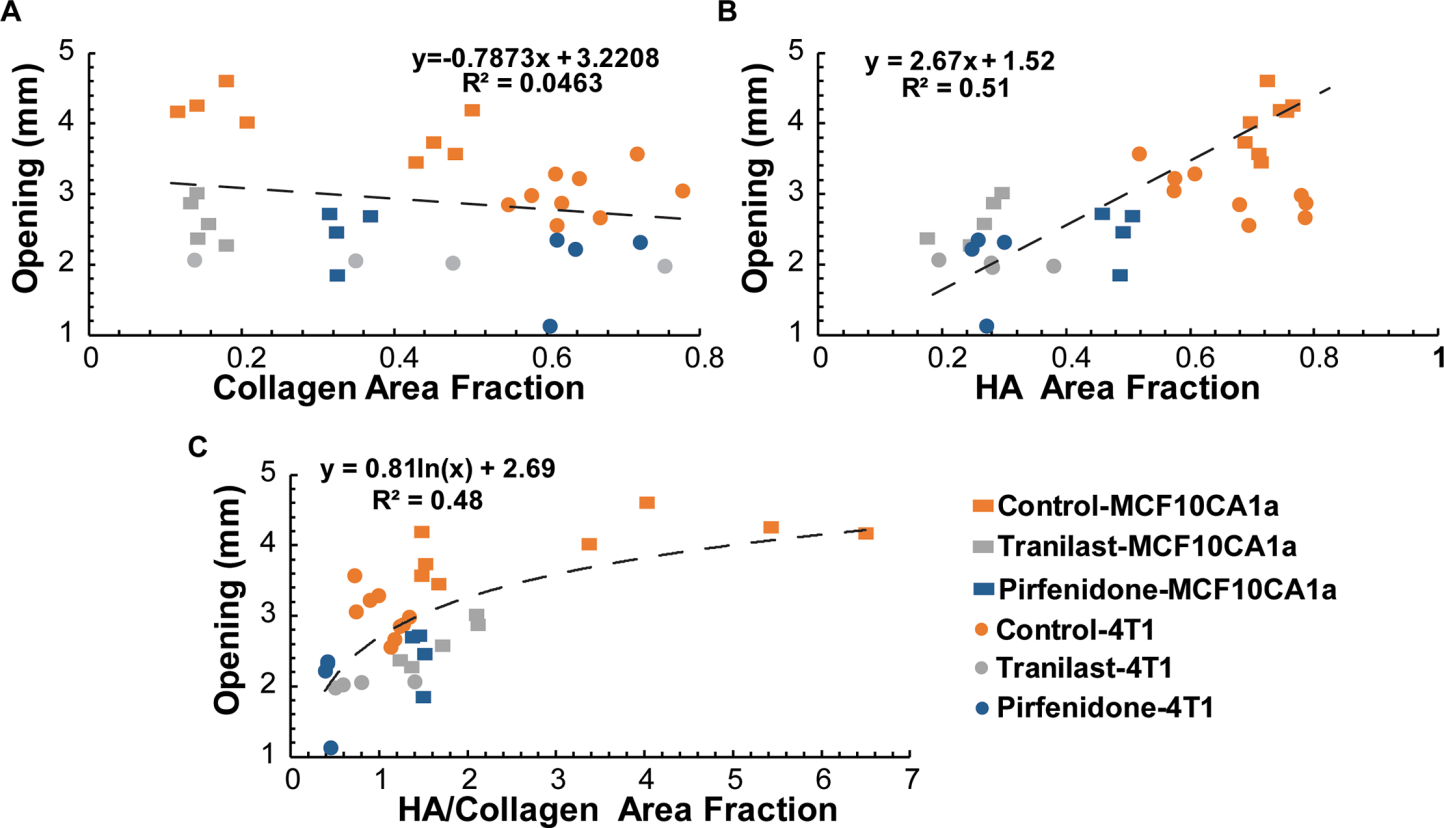
Effect of ECM composition on tumor opening. (**A**) Collagen area fraction does not correlate with opening, whereas there is a correlation of tumor opening with hyaluronan (HA) area fraction (**B**) and the ratio of HA/collagen area fraction (**C**). Five tumor specimens (n = 5) from each tumor type were used.

To investigate any potential correlation between the accumulation of growth-induced stress with the extracellular matrix structure, we plotted in Fig 4 the tumor opening as a function of collagen or hyaluronan area fraction and also as a function of the ratio of the two. Even though there was no correlation between tumor opening and collagen content, there was a good linear correlation with hyaluronan area fraction and a partial correlation with the ratio of hyaluronan to collagen. These results indicate the critical role of hyaluronan in the accumulation of mechanical forces in tumors, further supporting the relationship between growth-induced and swelling stress [11].

### Growth-induced stress is related to hyaluronan content and tissue stiffness

**Fig 5:**
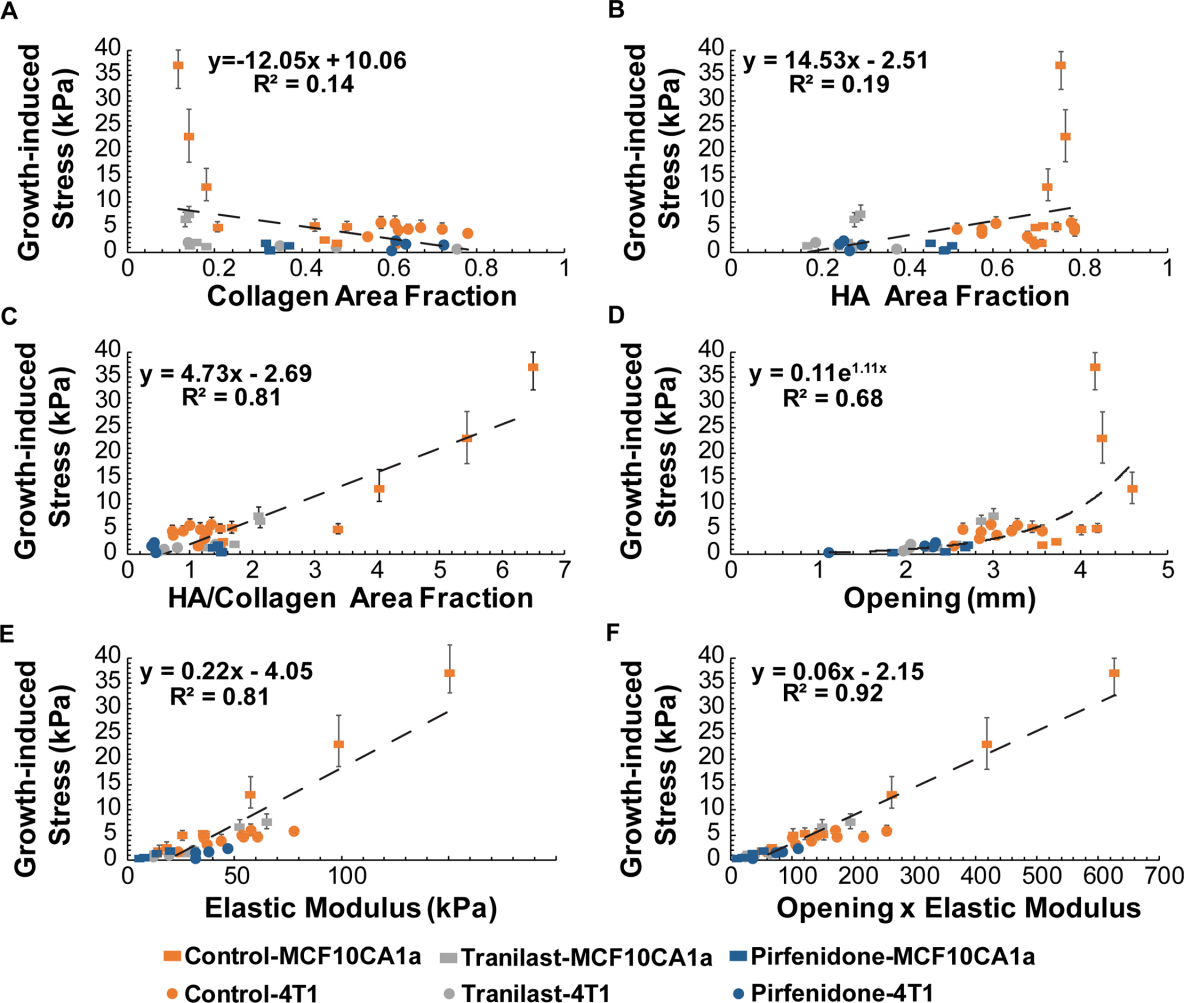
Effect of ECM composition and mechanical properties on growth-induced stress. (**A**) Collagen or (**B**) hyaluronan (HA) area fraction is not associated with growth-induced stress, whereas a relation seems to exist when (**C**) the ratio of HA/collagen area fraction is employed. (**D**) Growth-induced stress does not depend on tumor opening but there is a good correlation between growth-induced stress and tumor elastic modulus (**E**) as well as with the product of tumor opening and elastic modulus (**F**). Error bars represent the range of estimated growth-induced stress.

To estimate the growth-induced stress from the tumor opening measurements, we simulated the experimental procedure using the elastic modulus as measured for each specimen separately. Fig 5 presents the growth-induced stress as a function of tumor content as well as tumor opening and elastic modulus. There is a dependence of growth-induced stress on hyaluronan to collagen area fraction (Fig 5C). Additionally, there is a good correlation of growth-induced stress with the tumor opening (Fig 5D) and the elastic modulus of the tissue (Fig 5E) and an excellent correlation with the product of the two (Fig 5F), implying that tissue stress relaxation follows a linear elastic behavior. Furthermore, our results suggest that even though measurements of tumor opening are simple and easy to be performed, they cannot be directly used for comparison of growth-induced stress between tumor types, without taking into consideration the elastic properties of the tumors.

### Swelling stress is comparable to growth-induced stress levels

**Fig 6:**
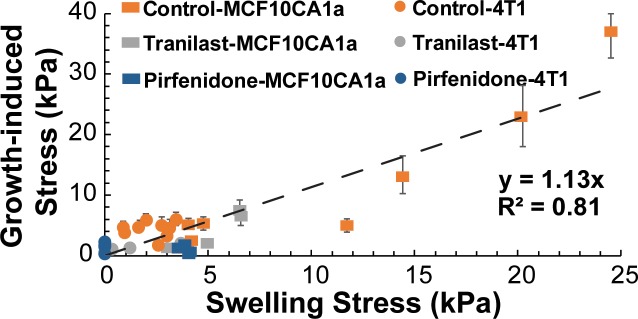
Swelling stress agrees well with growth-induced stress. The good correlation of the two types of stress is given by the good fit along the y = x dash line. Error bars represent the range of estimated growth-induced stress.

Using our previous analysis [11], we calculated the swelling stress from the ratio of hyaluronan to collagen content (Eq 4) and compared the values with the growth-induced stress levels (Fig 6). Interestingly, there is a good correlation between the two types of stress that fits well along the y = x line (Fig 6). Taking collectively our results into account we can conclude that growth-induced stress and swelling stress levels are comparable.

### Parametric analysis of geometrical and mechanical parameters for the calculation of growth-induced stress

Geometrical parameters that affect calculation of the tumor opening experiment are the thickness of the cut as well as the thickness of the peripheral capsule. For the baseline simulations, we took the thickness of the cut to be 80% of the tumor thickness and the thickness of the capsule 10% of that of the tumor according to our experimental protocol and previous research [7]. Additionally, the relative stiffness of the capsule to the stiffness of the tumor might also affect tumor opening. In our simulations, we assumed the peripheral capsule to be 10 times stiffer than the inner tumor region [7]. To test how the values of these parameters affect our calculations of growth-induced stress, we performed a parametric analysis. As shown in Figure F in S1 File, the thickness of the cut as well as the stiffness of the capsule have a minor or small effect on tumor opening, whereas the thickness of the capsule compared to the tumor size is more critical in our calculations of growth-induced stress. However, histological analysis has shown that the thickness of this capsule, which is a result of remodeling of peripheral collagen fibers, does not exceed 10% of tumor thickness. Therefore, any change in the values of the parameters considered here is not expected to affect the conclusions of our study.

## Discussion

Despite the crucial role that mechanical forces play in tumor growth and therapy, the study of the biomechanical behavior of solid tumors remains a relatively unexplored area of research as least compared to the biomechanics of other connective tissues, such as blood vessel, heart, tendons and articular cartilage [4,6]. The existence of growth-induced/residual stress in solid tumors was suspected for more than two decades but it was not until 2012 that we systematically studied its causes, consequences and remedies [7,8]. Furthermore, we and co-workers employed this simple tumor opening experiment as a technique to test the ability of common drugs to alleviate mechanical forces in tumors in order to improve the delivery of chemotherapy and nanomedicines by decompressing tumor blood vessels. Such drugs include: losartan (Cozaar^®^, Merck Pharmaceuticals) a common anti-hypertensive drug, pirfenidone (Esbriet^®^, Roche) approved for the treatment of idiopathic pulmonary fibrosis and tranilast (Rizaben^®^, Kissei Pharmaceuticals) approved in Japan and South Korea as an anti-allergic and anti-fibrotic drug [11,17,18,27,28]. Regarding the swelling behavior of tumors, it was only recently that the swelling of cancer cells and the hyaluronan-derived swelling of tumors was systematically studied [11,29].

Here, using a series of experimental data and computational calculations, we found that even though tumor opening is related to hyaluronan content (Fig 4B), growth-induced stress is more closely related to the ratio of hyaluronan to collagen area fraction (Fig 5C), and that it is affected by tumor stiffness (Fig 5E). This can be explained by the fact that—as it was mentioned in the Introduction—the opening of the tumor is due to the bulging of the intratumoral region, which is mainly caused by hyaluronan swelling. Whereas the compressive growth-induced stress at the interior of the tumor—that causes the bulging of the inner tumor region and it is due to hyaluronan—is balanced by the tensile growth-induced stress at the periphery that causes the retraction of the tumor surface and it is due to the peripheral collagen capsule [6,7]. Therefore, both hyaluronan and collagen should determine growth-induced stress. Interestingly, even though both cancer cell lines employed in this study had similar levels of hyaluronan, the difference in collagen content, and thus, in the hyaluronan to collagen fraction resulted in significantly different levels of growth-induced stress. The same was also observed for the swelling stress because even though swelling is caused by hyaluronan, it is restricted by the collagen fibers of the tumor ECM.

Therefore, stress-alleviating drugs targeting both collagen and hyaluronan can alleviate intratumoral solid stresses both by reducing ECM content and by making the tumor less stiff [11,17,18,27]. Our findings that relate the mechanical state of a tumor to data derived from histological analysis can be used for better understanding of the mechano-pathology of cancer and for the design of stress-alleviating therapeutic strategies. For instance, a similar to this study histological analysis could be easily performed in human tumor biopsies and provide insights on the magnitude of mechanical stresses developed within the tissue. Also, it could further suggest if targeting of collagen, hyaluronan or both would be more beneficial in alleviating these stresses.

## Supporting information

S1 File Text of Figures A-F.
(PDF)

S2 File Text of dataset. (PDF)
