# Supplementary material for: Accumulation of mechanical forces in tumors is related to hyaluronan content and tissue stiffness
Source: PLoS One. 2018 Mar 21;13(3):e0193801. doi: 10.1371/journal.pone.0193801 (PMC5862434; doi:10.1371/journal.pone.0193801)
Supplement: S2 File — (PDF) [file pone.0193801.s002.pdf]

	Opening (mm)					
	MCF10CA1a			4T1		
	Control	Tranilast	Pirfenidone	Control	Tranilast	Pirfenidone
**Average**	3.99625	2.62	2.43	3.003333	2.0275	2.005
**SE**	0.136786	0.141633	0.201866	0.106197	0.01797	0.292987
**Over All Anova**						
**F**	32.93			15.65		
**P value**	< 0.0001			0.0003		
**Tukey's multiple comparisons test**						
**Control vs. TRanilast**	< 0.0001	****		0.0014	**	
**Control vs. Pirfenidone**	< 0.0001	****		0.0012	**	

	Area Fraction					
	MCF10CA1a/collagen			4T1/collagen		
	Control	Tranilast	Pirfenidone	Control	Tranilast	Pirfenidone
**Average**	0.32625	0.15	0.3375	0.642222	0.43	0.6425
**SE**	0.053584	0.008944	0.011086779	0.023792	0.127606	0.027195281
**Over All Anova**						
**F**	4.899			3.939		
**P value**	0.0244			0.0439		
**Tukey's multiple comparisons test**						
**Control vs. TRanilast**	0.0319	*		0.0454	*	
**Control vs. Pirfenidone**	0.9842	ns		> 0.9999	ns	

	MCF10CA1a/HA			4T1/HA		
	Control	Tranilast	Pirfenidone	Control	Tranilast	Pirfenidone
	0.72875	0.256	0.4875	0.668889	0.2825	0.27
	0.010253	0.02064	0.010307764	0.034579	0.03881	0.010801234
	311.8			43.32		
	< 0.0001			< 0.0001		
	< 0.0001	****		< 0.0001	****	
	< 0.0001	****		< 0.0001	****	

	Elastic Modulus					
	MCF10CA1a			4T1		
	Control	Tranilast	Pirfenidone	Control	Tranilast	Pirfenidone
**Average**	63.22625	27.52	11.7525	52.62889	23.395	34.65
**SE**	11	3.969055	3.188303	4.049433	4.727983	1.688481
**Over All Anova**						
**F**	4.763			12.34		
**P value**	0.0264			0.0008		
**Tukey's multiple comparisons test**						
**Control vs. TRanilast**	0.1206	ns		0.0009	***	
**Control vs. Pirfenidone**	0.0323	*		0.029	*	

	Grwth-induced stress					
	MCF10CA1a			4T1		
	Control	Tranilast	Pirfenidone	Control	Tranilast	Pirfenidone
**Average**	14.96875	3.54	0.9925	4.361111	1.2625	1.4375
**SE**	0.89	0.374967	0.342135	0.442042	0.294657	0.419014
**Over All Anova**						
**F**	6.129			15.64		
**P value**	0.0122			0.0003		
**Tukey's multiple comparisons test**						
**Control vs. TRanilast**	0.044	*		0.001	**	
**Control vs. Pirfenidone**	0.0217	*		0.0017	**	

	Swelling stress					
	MCF10CA1a			4T1		
	Control	Tranilast	Pirfenidone	Control	Tranilast	Pirfenidone
**Average**	14.435	4.122	3.9075	2.263333	0.8325	0.3425
**SE**	1.2	0.736868	0.127238	0.314002	0.402127	0.049392
**Over All Anova**						
**F**	5.512			9.552		
**P value**	0.0172			0.0024		
**Tukey's multiple comparisons test**						
**Control vs. TRanilast**	0.0353	*		0.0263	*	
**Control vs. Pirfenidone**	0.0458	*		0.0037	**	
