# Supplementary material for: Accumulation of mechanical forces in tumors is related to hyaluronan content and tissue stiffness
Source: PLoS One. 2018 Mar 21;13(3):e0193801. doi: 10.1371/journal.pone.0193801 (PMC5862434; doi:10.1371/journal.pone.0193801)

Supporting information

Supporting figures

Figure A. Representative stress-strain curve of the confined compression experiment.

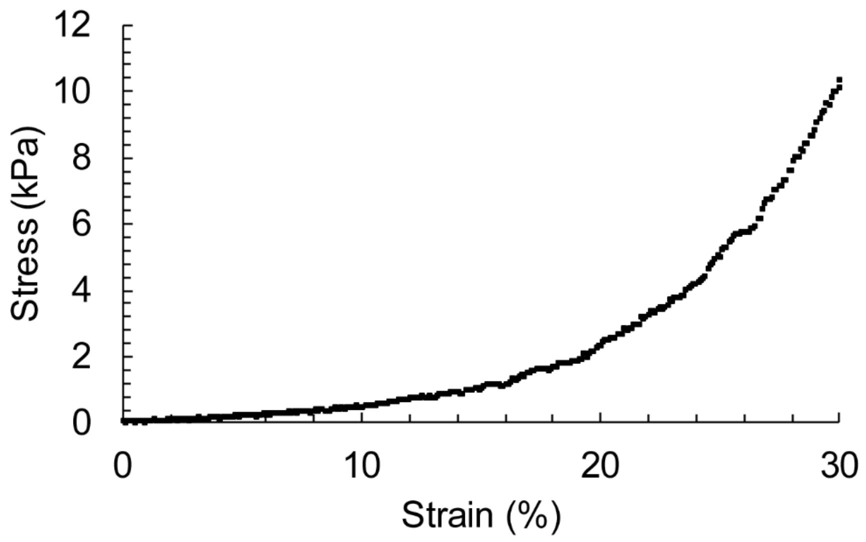

Figure B. Boundary Conditions employed by the computational model for calculations of growth-induced stress. Schematic of the tumor domain, we modeled the one-quarter of the domain assuming spherical symmetry. The applied boundary are: stress free at the free surface of the tumor and symmetry at the planes of symmetry. The schematic also shows the two different regions: intratumoral and peripheral region.

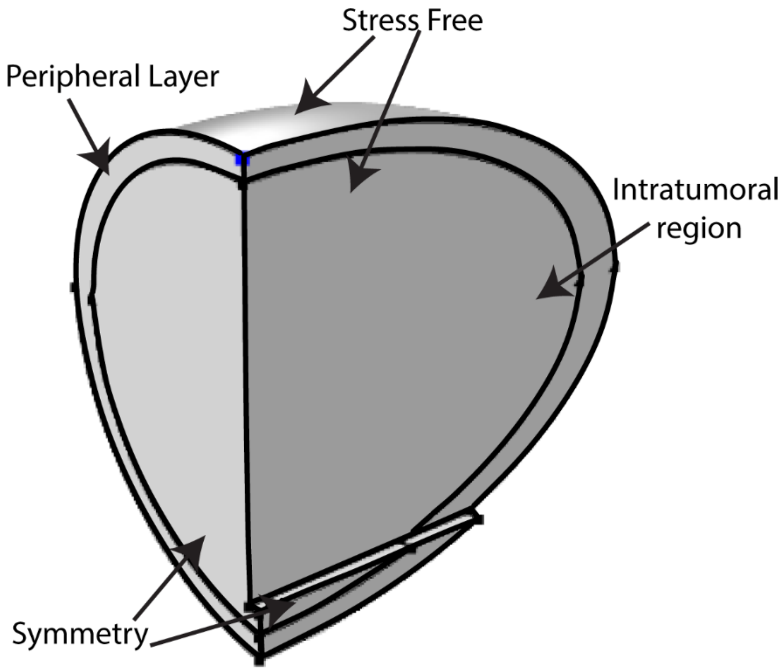

Figure C. Mesh-independence analysis of the finite elements model. The number of elements varied from 4,079 to 526,467 elements and the predicted by the model tumor opening was used for comparison.

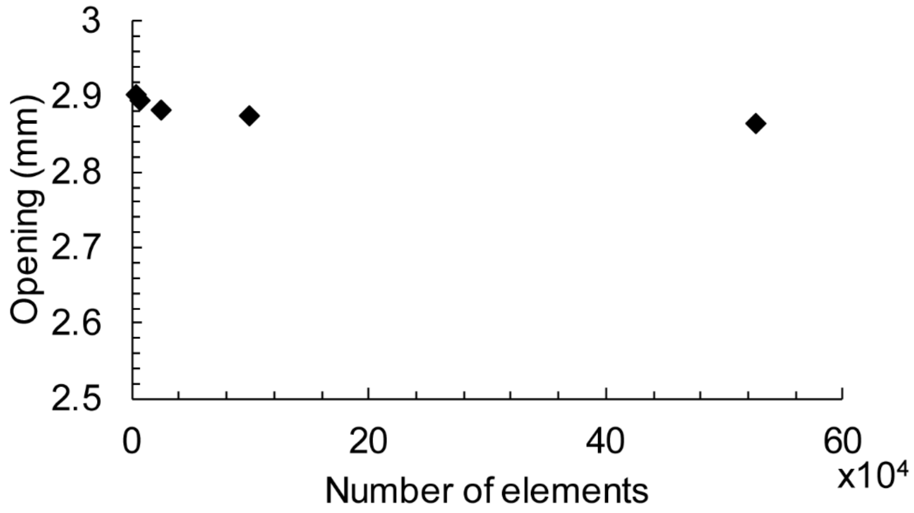

Figure D. Measurement of the thickness of the periphery layer. Representative samples of immunofluorescence staining for collagen and dapi showing the peripheral layer of the tumors (left). Image analysis was used to calculate the relative thickness of the peripheral collagen layer (capsule) and results are presented in the box plot (n=8). Scale bar:500μm.

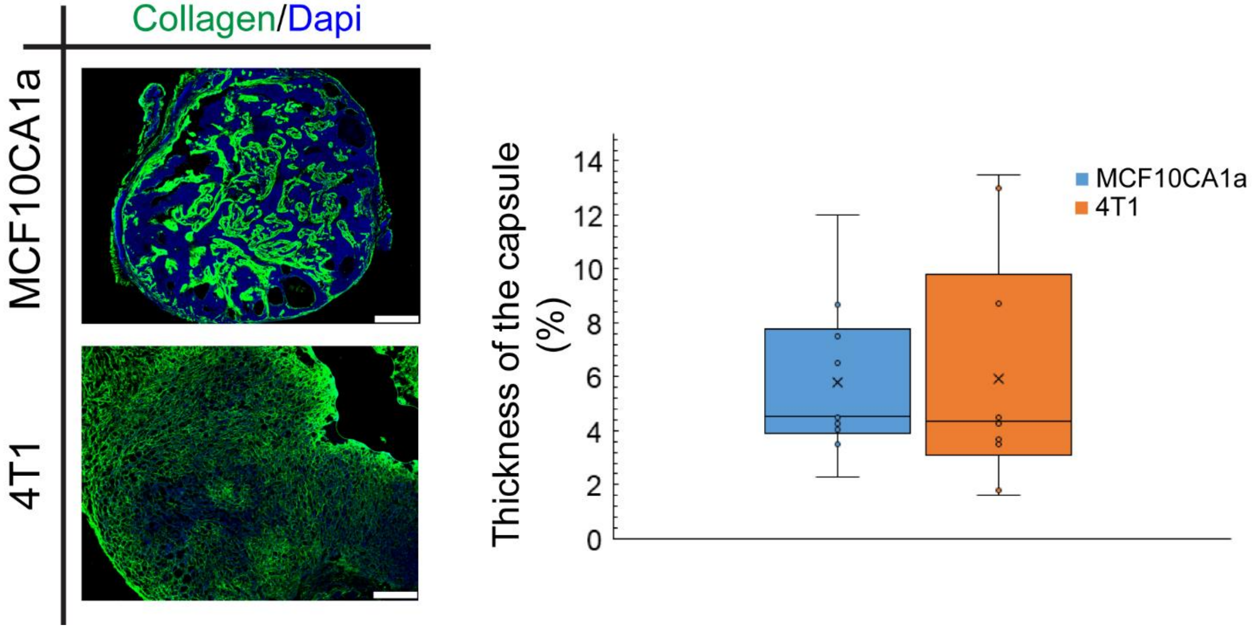

Figure E. Representative immunofluorescence staining for collagen, hyaluronan (HA) and Dapi. The different compositions and organization is shown. Scale bar 100 μm

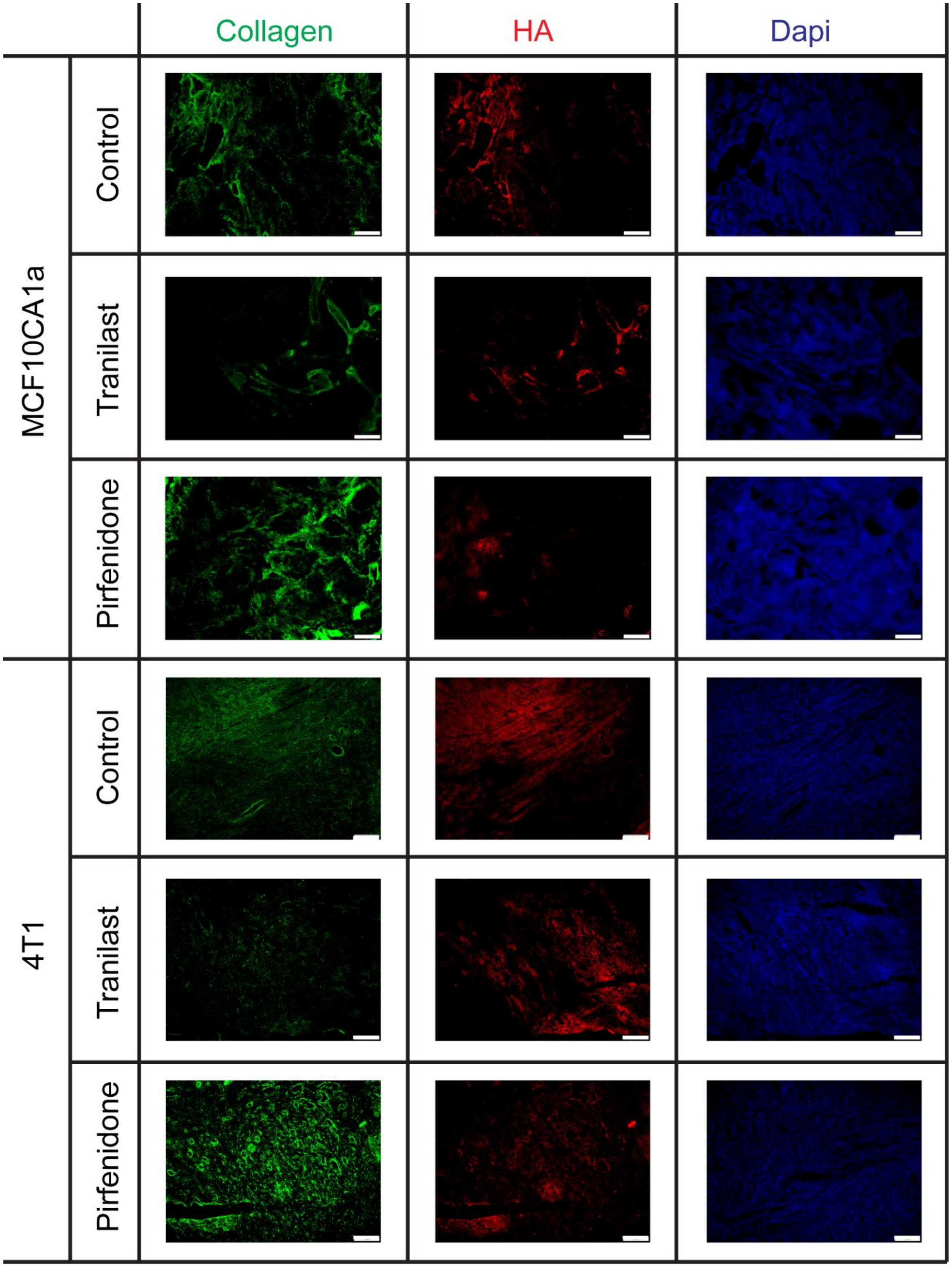

Figure F. Results of the parametric analysis of the computational model. The plots show tumor opening calculations as a function of (A) thickness of the cut (percent to tumor diameter), (B) thickness of the capsule (percentage of tumor diameter) and (C) capsule stiffness (ratio of capsule shear modulus to tumor shear modulus).

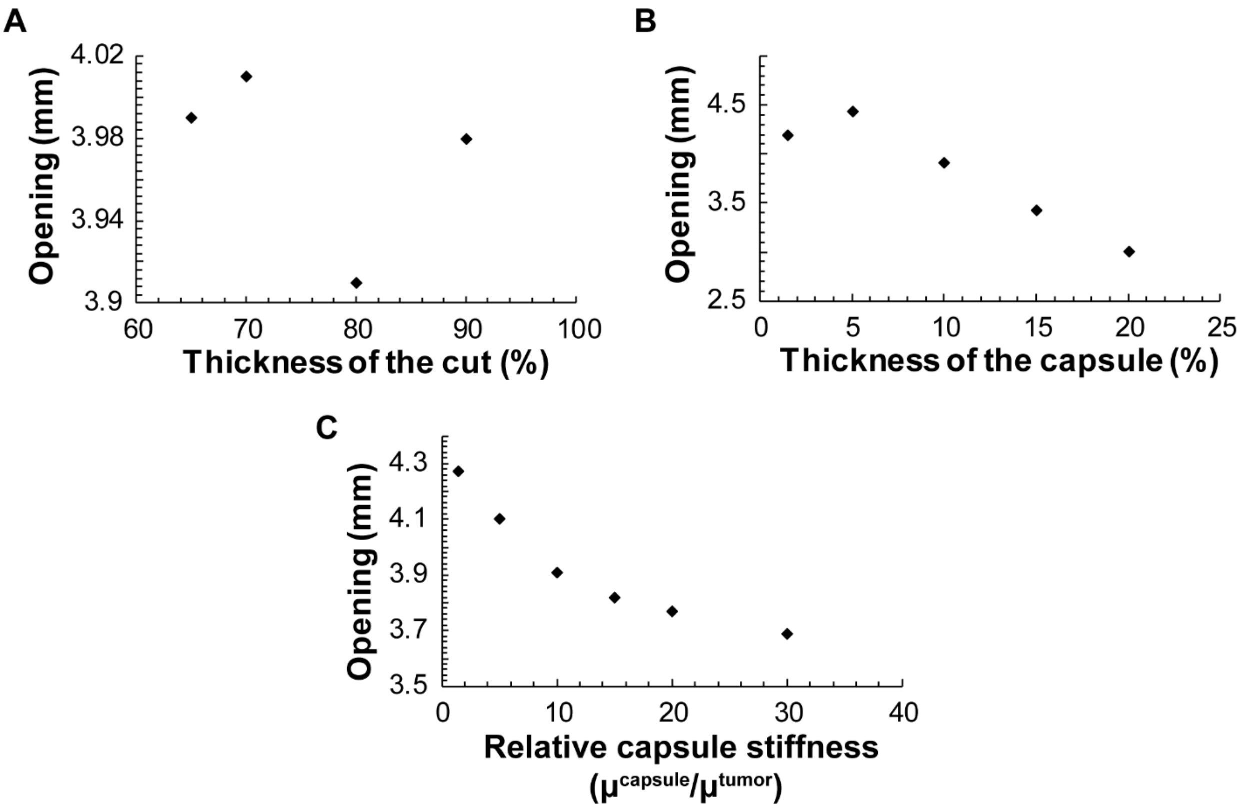

Supplement: S1 File — Text of Figures A-F. (PDF) [file pone.0193801.s001.pdf]
